# Pulmonary Visceral Pleura Biomaterial: Elastin- and Collagen-Based Extracellular Matrix

**DOI:** 10.3389/fbioe.2022.796076

**Published:** 2022-03-30

**Authors:** Xiao Lu, Ling Han, Ghassan S. Kassab

**Affiliations:** California Medical Innovations Institute, San Diego, CA, United States

**Keywords:** lung, structure, compliance, biomechanics, biocompatibility, nerve, skin

## Abstract

**Objective:** The goal of the study is to determine the structural characteristics, mechanical properties, cytotoxicity, and biocompatibility of the pulmonary visceral pleura (PVP).

**Background:** Collagen and elastin are the major components of the extracellular matrix. The PVP has an abundance of elastin and collagen that can serve as a potential biomaterial for clinical repair and reconstructions.

**Methods:** The PVP was processed from swine and bovine lungs. Chemical analyses were used to determine collagen and elastin contents in the PVPs. Immunofluorescence microscopy was used to analyze the structure of the PVP. The stress–strain relationships and stress relaxation were determined by using the planar uniaxial test. The cytotoxicity of the PVP was tested in cultured cells. In *in vivo* evaluations, the PVP was implanted in the sciatic nerve and skin of rats.

**Results:** Collagen and elastin contents are abundant in the PVP with larger proportions of elastin than in the bovine pericardium and porcine small intestinal submucosa. A microstructural analysis revealed that the elastin fibers were distributed throughout the PVP and the collagen was distributed mainly in the mesothelial basal lamina. The incremental moduli in stress–strain curves and relaxation moduli in the Maxwell–Wiechert model of PVP were approximately one-tenth of the bovine pericardium and small intestinal submucosa. The minimal cytotoxicity of the PVP was demonstrated. The axons proliferated in the PVP conduit guidance from proximal to distal sciatic nerves of rats. The neo-skin regenerated under the PVP skin substitute within 4 weeks.

**Conclusions:** The PVP is composed of abundant collagen and elastin. The structural characteristics and mechanical compliance of the PVP render a suitable biological material for repair/reconstruction.

## Introduction

Biological tissues have several important advantages for prostheses over synthetic materials. The primary benefits include rapid and complete endothelialization, higher resistance to infection, and overall improved biocompatibility (Badylak et al., 1995; Lindberg and Badylak, 2001; Tamariz and Grinnell, 2002; Maestro et al., 2006; Waterhouse et al., 2011; Gauvin et al., 2013; Walters and Stegemann, 2014; Sánchez et al., 2018; Lu et al., 2019; Lu et al., 2020). In biological tissues, collagen and elastin are two major components of the extracellular matrix (ECM). Collagen is the most abundant ECM protein and provides a highly biocompatible environment for cells. This high biocompatibility makes collagen a perfect biomaterial for implantable medical products and scaffolds for *in vitro* testing systems (Meyer, 2019). Elastin is also abundant in ECM and highly biocompatible for cellular proliferation. In comparison with collagen, elastin can render ECM more resilience due to its molecular coil structure. Elastin has advanced durability over collagen due to its resistance to proteases (Shapiro et al., 1991; Mecham, 2018). In biocompatibility, RGD (arginine–glycine–aspartate) in collagen is the dominant domain for cellular adhesion and proliferation (Tamariz and Grinnell, 2002; Walters and Stegemann, 2014). Specific domains in elastin, instead of RGD, however regulate cellular proliferation (Daamen et al., 2007; Waterhouse et al., 2011), which differentiates the function of elastin. In fact, both collagen and elastin are essential for the cellular environment and homeostasis. Therefore, the merits of both collagen and elastin ought to be brought into the ECM biomaterials.

The bovine pericardium (bPcm) and swine small intestinal submucosa (SIS) are popular biomaterials and broadly utilized in clinical practice. The bPcm and SIS are defined as collagen-based EMC since collagen is the dominant component and elastin is relatively minor (Badylak et al., 1995; Lindberg and Badylak, 2001; Maestro et al., 2006; Gauvin et al., 2013). Therefore, the ameliorations of elastin are insufficiently utilized in bPcm and SIS. It is known that the proportions of collagen and elastin are diverse in the ECM of various organs (Daamen et al., 2007; Meyer, 2019). A larger proportion of elastin in the ECM can result in more resilience. The lung is known as an elastic organ, and the proportion of elastin is much larger in the lung than in the bPcm and SIS (Oldmixon and Hoppin, 1984; Fraser et al., 2005; Mecham, 2018). Hence, the lung tissue ECM (e.g., pulmonary visceral pleura, PVP) includes the benefits of both collagen and elastin as a biomaterial. The PVP is a serous membrane that closely sheathes the surface of the lung (Oldmixon and Hoppin, 1984; Humphrey et al., 1986; Fraser et al., 2005). The PVP maintains key roles in preserving lung function (Ligas et al., 1984; Michailova, 1997; Fraser et al., 2005). The cyclical inflation and deflation during respiration tensions and relaxes the PVP and thereafter renders the potential of the PVP to resist cyclic deformation.

Despite the potentially advantageous properties, a detailed characterization of the PVP as an ECM biomaterial has not been previously described. The role for the PVP as an ECM biomaterial remains largely unexplored. Thus, we studied the structural and mechanical properties of swine and bovine PVP. The cytotoxicity and biocompatibility of the PVP were evaluated. The studies of *in vivo* implant were performed to identify the potential for the PVP as a novel biomaterial in tissue repair/reconstruction.

## Materials and Methods

### Tissue Preparation

**FIGURE 1:**
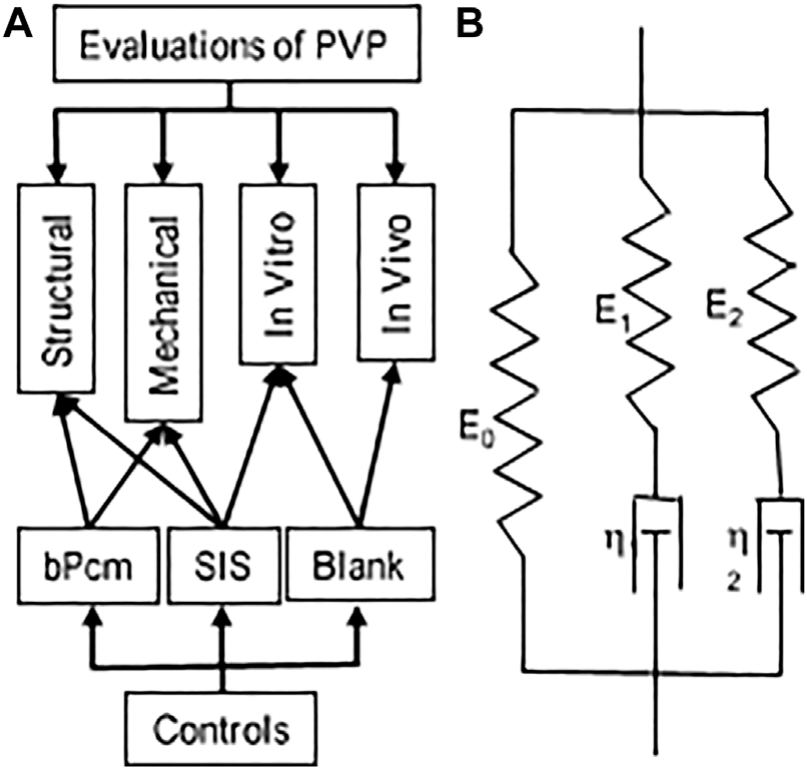
Schematic representations of the evaluation and Maxwell–Wiechert model. **(A)** An overall picture of the evaluations. **(B)** Two Maxwell elements with moduli *E*_*1*_ and *E*_*2*_ and viscosities *η*_*1*_ and *η*_*2*_ provide a model with two relaxation times *τ*_*1*_ and *τ*_*2*_, respectively, that is, a fast-response element (*E*_*1*_ and *τ*_*1*_) and a slow-response element (*E*_*2*_ and *τ*_*2*_).

Swine lung (*n* = 6), bovine lungs (*n* = 6), bovine pericardium (*n* = 6), and swine small intestine (*n* = 6) were obtained from a local abattoir and transported in cool saline. All organs and tissues were processed within 4 h post-harvest. The PVP was isolated *via* blunt dissection from the pulmonary parenchyma. Following isolation of sections from the posterior lobe, the specimens were laid flat and rinsed with 4°C saline. The specimens of bovine pericardium (bPcm) were rinsed with 4°C saline. The specimens of swine small intestinal submucosa (SIS) were processed by removing the mucosa and muscle layers from the small intestine and then rinsed with 4°C saline. The overall approach is represented in Figure 1A. The specimen from each animal was cut with scissors into three samples, which were assigned to categories 1, 2, and 3 for various experiments, as given in Table 1. Briefly, the samples in category 1 were directly stored in cool saline for the analyses of collagen and elastin contents. The samples in category 2 were laid flat and fixed using 4% paraformaldehyde for immunofluorescence microscopy. The samples in category 3 were cross-linked in 0.625% buffered glutaraldehyde solution at 23°C for 24 h, and then divided into three pieces for mechanical tests, *in vitro* toxicity, and *in vivo* biocompatibility, respectively. Glutaraldehyde cross-link is routine to minimize immune rejection for heterogenous implants.

**TABLE 1:** Distribution of various tissues for mechanical and biological tests.

	Category 1	Category 2	Category 3: 0.625% glutaraldehyde fixation			
	Ela and Col contents	Immunofluorescence	Mechanical test	*In vitro* toxicity	Biocompatibility in rats (*n = 12*)	
					Nerve, *n = 6*	Skin, *n = 6*
6 pigs, sPVP	*n = 6*	*n = 6*	*n = 6*	*n = 6*	*n = 6*	*n = 6*
6 pigs, SIS	*n = 6*	*n = 6*	*n = 6*	*n = 6*	--	--
6 cows, bPVP	*n = 6*	*n = 6*	*n = 6*	--	--	--
6 cows, bPcm	*n = 6*	*n = 6*	*n = 6*	--	--	--

### Collagen Content

The collagen analysis was based on the alkaline hydrolysis of samples to yield free hydroxyproline (Huszar et al., 1980). The released hydroxyproline is oxidized to form a reaction intermediate, which when further reacted forms brightly colored chromophore that can be detected at OD 550 nm (Kesava Reddy and Enwemeka, 1996). The collagen assay kit was obtained from Abcam (Total Collagen Assay Kit ab222942, Abcam, US) (da Silva et al., 2015). The tissue samples were prepared at 0.1–0.2 gm and hydrolyzed by alkaline solution. The assay kit employed a proprietary developer solution to accurately measure collagen in hydrolysates. The assay can detect as low as 0.5 µg collagen/well. The result was represented as micrograms (μg) of collagen per milligram (mg) of the tissue dry weight.

### Elastin Content

We employed the acid hydrolysis and alkaline treatment for elastin evaluation. The acid hydrolysis converts all elastin in the tissue to soluble elastin by oxalic acid (hydrolytic solvent) (Partridge et al., 1955). The soluble elastin (tropoelastin, 50–70 kDa) is the precursor protein molecules to elastin, which is synthesized by the cell and cross-linked into elastin fibrils during their export into the extracellular matrix. These cross-linked elastin fibrils associate with fibrillin-containing microfibrils to form the elastic fiber, which is an insoluble and durable complex (Daamen et al., 2007; Wise et al., 2009). The soluble elastin can be evaluated by chemiluminescence using the dye reagent 5,10,15,20-tetraphenyl-21H,23H-porphine tetra-sulfonate (TPPS). The dye reagent binds to the “basic” and “non-polar” amino acid sequences found in mammalian tropoelastin (Kothapalli and Ramamurthi, 2009). The presence of other soluble proteins or complex carbohydrates does not interfere with the dye reagent. The commercialized kit (Fastin™ Elastin assay, Biocolor, UK) was used to evaluate the elastin in the tissues. The result was represented as micrograms (μg) of converted soluble elastin per milligram (mg) of the tissue dry weight.

The alkaline treatment is performed to use alkaline solvent to hydrolyze ECM, except elastin, for the evaluation of the insoluble elastin in the tissues. In alkaline treatment, the insoluble elastin in various tissues was retained due to its resistance to the hydrolysis during treatment with hot alkaline solution (Harkness et al., 1957; Wolinsky, 1970; Maestro et al., 2006). Briefly, the samples were trimmed to approximate 9 cm^2^ and delipidated in chloroform/methanol (1:1 v/v). The delipidated samples were dried in room temperature and soaked in the lysis buffer (0.3% sodium dodecyl sulfate) for 12 h to remove cell proteins in the samples (Neethling et al., 2003). The remnant was minced and immersed in 0.1M NaOH solution (v/v: ∼1/10), and heated at 100°C for 15 min. The ECM proteins except elastin were hydrolyzed by an alkaline solution, and the supernatant was discarded. The alkaline hydrolysis was repeated four times so that only insoluble elastin was retained in the remnant. The insoluble elastin was lyophilized. The dry insoluble elastin was weighed. The result was represented as micrograms (μg) of insoluble elastin per milligram (mg) of the tissue dry weight.

### Immunofluorescence Microscopy for Collagen and Elastin Visualization

The samples (width × length: ∼5 × 5 mm) were fixed in 4% paraformaldehyde overnight and subsequently sectioned with a cryostat. The sections were processed for immunofluorescence procedures; that is, blocking with phosphate-buffered solution (PBS, pH 7.4) contained 10% donkey serum for 1 h, permeabilization with PBS contained 0.25% Triton for 15 min, primary antibody incubation, and fluorescence secondary antibody incubation. The primary antibodies included anti-elastin (Cat. #sc17480, 1/30 dilution with PBS contained 0.25% Triton and 2.5% donkey serum, Santa Cruz Biotechnology) and collagen (Cat. #ab7778, 1/2530 dilution with PBS contained 0.25% Triton and 2.5% donkey serum, & ab6586, 1/20 dilution with PBS contained 0.25% Triton and 2.5% donkey serum, Abcam). The primary antibodies were incubated at 4°C overnight. The fluorescent dye-conjugated secondary antibodies (Cat. #A10040, A10036, A11081, & A11058, 1/100 dilution with PBS contained 0.25% Triton and 2.5% donkey serum, Thermo Fisher Scientific) were incubated at room temperature (22°C) for 1 h. The fluorescence reagent (Cat. #Hoechst 33342, 1/5000 dilution with PBS contained 0.25% Triton and 2.5% donkey serum, Life Technology) was used for cellular nuclei visualization. The fluorescent dyes were visualized using a fluorescence microscope (Eclipse Ts2R, Nikon).

### Planar Uniaxial Stress–Strain Relation and Stress Relaxation

All samples for testing were trimmed to the dimensions of 22 × 55 mm (width × length) sheets. The thickness of the samples was measured in a no-load state. The samples were then secured in the clamps of a uniaxial device (Mark-10, NY). The loading rate was selected at 1.7 mm/s. The maximum stretch ratio for each sample was 1.5. The precondition was performed until repeatable loading forces were obtained (Zhao et al., 2014). The force–displacement relation was obtained from the loading curve following the precondition. The Cauchy stress and strain were computed to determine the stress–strain curve. In the stress relaxation test, the samples were elongated to a Cauchy strain of 0.3 at a speed of 3.3 mm/s. The stresses gradually decreased to a stable value in a period of approximate 300 s. We employed the Maxwell–Wiechert model for the analyses of viscoelastic properties of the tissues (Figure 1B). The relaxation modulus as a function of time for the Maxwell–Wiechert model can be expressed as follows:

σ(t)ε0=ERelax (t)=E0+E1 exp(−tτ1)+E2 exp(−tτ2),

where *σ*(*t*) is stress (Cauchy stress) relaxation with time, *ε*_*0*_ is the constant Cauchy strain during the stress relaxation test, *E*_*relax*_(*t*) is the time-dependent elastic modulus, and *E*_*0*_ is the time-independent elastic modulus. The relaxation time *τ*_*i*_ of each Maxwell element is equal to the coefficient of viscosity of the dashpot (*η*_*i*_) divided by the elastic modulus of the spring (*E*_*i*_). The elastic modulus and relaxation times were obtained by fitting the stress–time data with the Maxwell–Wiechert model.

### *In Vitro* Cytotoxicity

The samples of sPVP (*n* = 6) and SIS (*n* = 6) were rinsed, transferred to serum-free media, and placed into a shaking incubator at 37°C for 24 h. The samples were cut into 15 mm discs and placed into individual wells of a 24-well plate, in which the two wells coated with fibronectin served as blank. The wells were filled with full media (DMEM with 10% sterile filtered fetal bovine serum, 1% L-glutamine, and 1% penicillin/streptomycin). Confluent NIH 3T3 cells were released from tissue culture plates using 0.25% trypsin, spun, resuspended, and counted. Overall, 100,000 cells were added to each well in fresh full media and incubated at 37ºC in 5% CO_2_ for 1 day. Non-adherent cells were discarded. The wells containing adherent cells were filled with fresh full media and incubated at 37ºC in 5% CO_2_ for 5 days. The adherent cells on samples were released with 0.25% trypsin, spun, and resuspended in 1 ml Dulbecco’s phosphate-buffered saline for cell counting. Fluorescent probes Calcein AM (ex/em: 485/530 nm) and ethidium homodimer (ex/em: 530/645 nm) were used to label living and dead cells. Reagents (LIVE/DEAD Viability/Cytotoxicity Kit, Invitrogen) were added in the cell suspension for the concentrations of 2 μM calcein AM and 4 μM ethidium homodimer. The suspension with the reagents was incubated at room temperature for 40 min. The fluorescent labeled cell suspensions were loaded to a hemocytometer (Bright-Line, Hausser Scientific). The number of living and dead cells in the hemocytometer was determined by counting using a fluorescence microscope (Ts2R, Nikon). Cell viability was calculated for the percentage of living cells in total cells (living plus dead cells).

### Ethics Statement

We used rodent models to examine the biocompatibility of the PVP. All experimental procedures and protocols used in the investigations were approved by the Institutional Animal Care and Use Committee in accordance with the Guide for the Care and Use of Laboratory Animals. All protocols regarding the use of animals in the research were approved by California Medical Innovations Institute IACUC. The approved protocol numbers were CalMI2-013 and CalMI2-015.

### *In Vivo* Evaluation as Nerve Guidance Conduit and Skin Substitute

Six swine PVPs (sPVPs) were constructed as hollow conduits (*n* = 6) to accommodate nerve regeneration in rodent sciatic nerve transection models (*n* = 6). Six Lewis rats of ages 5–6 months were anesthetized with 1.8% isoflurane. The hindquarters of rats were shaved and sterilized. The skin was cut parallel to the femur, and the sciatic nerve was exposed by means of a gluteal muscle slitting incision. With the help of a dissecting microscope, the sciatic nerve was excised over a span of approximate 12 mm between proximal and distal stumps. The PVP elastin conduit was placed near the two stumps of the sciatic nerve. The nerve stumps were inserted into the conduit and anchored to the conduit by means of 2–3 fine stiches with 10–0 monofilament suture. The proximal and distal nerve fibers were tension-free, while the conduit was implanted. The muscle was closed with running 6–0 absorbable suture, and the skin was closed with 5–0 prolene suture. Buprenorphine HCl (0.01–0.05 mg/kg) was administrated for postoperative pain control. The sciatic functional index (SFI) was evaluated biweekly. At the terminal study in 12 weeks, the distal muscle reinnervation was assessed. The contractile forces of the gastrocnemius muscle were measured during the tetanic stimulation (4 V, 100 Hz) by a bipolar electrode placed at the proximal end of the sPVP nerve guidance conduit. After a full rest, the bipolar electrode was moved to the distal end of the sPVP nerve guidance conduit. The contractile forces of the gastrocnemius muscle were measured again during the tetanic stimulation. The bipolar electrode was then placed on the contralateral non-traumatic sciatic nerve, and the tetanic contraction of the collateral gastrocnemius muscle was measured. The ratio of the ipsilateral tetanic contractions of sPVP nerve guidance conduit to the contralateral tetanic contractions was calculated. The ipsilateral and contralateral gastrocnemius muscles were weighed after euthanasia, and the weight ratio was calculated. The sPVP nerve guidance conduit and contralateral non-traumatic sciatic nerve were harvested for histologic and immunofluorescence analyses. The tissues were fixed with 4% paraformaldehyde and sectioned with a cryotome. Hematoxylin and eosin (HE) staining was performed. The sections were processed for immunofluorescence. The primary antibodies included anti-NF-L (neurofilament light polypeptide, Cat #: sc71678, 1/30 dilution with PBS contained 0.25% Triton and 2.5% donkey serum, Santa Cruz Biotechnology) and anti-MBP (myelin basic protein, Cat #: sc376995, 1/30 dilution with PBS contained 0.25% Triton and 2.5% donkey serum, Santa Cruz Biotechnology). The fluorescent secondary antibodies were visualized using a fluorescence microscope (Ts2R, Nikon). Each section was imaged by division into four squares. The myelinated axonal number and width in each division were counted and measured using image tool (ImageJ), respectively. The total number of myelinated axons in each section was the sum of the number in the four squares. The average of myelinated axonal width was calculated in the four squares. The mean and standard deviation were calculated from the data of six rats.

The sPVP was also used as skin substitute of an incised wound in rodent models (*n* = 6). Six Wistar rats of ages 5–6 months were anesthetized with 1–2% isoflurane. The hair on the dorsum was removed, and the skin was disinfected with Nolvasan, Betadine, and 70% alcohol. A piece of 2 × 4-cm dorsal skin was surgically excised to create a wound for repair. The sPVP was carefully covered on the wound and sutured to the adjacent skin. Then, an antiseptic bandage was covered to protect the sPVP on the wound from infections. The rats survived for 4 weeks postoperation. The neo-dermal tissues were harvested after euthanasia. The tissues were fixed with 4% paraformaldehyde and sectioned with a cryotome. The HE stains were performed. The sections were processed for immunofluorescence. The primary antibodies included anti-collagen IV (Cat #: ab6586, 1/20 dilution with PBS contained 0.25% Triton and 2.5% donkey serum, Abcam) and anti-sialic acid (wheat germ agglutinin, Cat #: W11261, 1/100 dilution with PBS contained 0.25% Triton and 2.5% donkey serum, Thermo Fisher Scientific). The fluorescent secondary antibodies were visualized using a fluorescence microscope (Ts2R, Nikon).

Statistical analysis: The data were presented as mean ± SD, and significant differences between two groups were determined by Student’s *t*-test (two-tailed distribution, two-sample unequal variance). Analysis of variance (ANOVA) was used to analyze the significant differences of mechanical tests among groups. A probability of *p* < 0.05 was indicative of a statistically significant difference.

## Results

### Contents of Elastin and Collagen

The contents of collagen and elastin in the various tissues are summarized in Table 2. The collagen contents in bPcm and SIS were significantly higher than those in the bPVP and sPVP (*p* < 0.05). The elastin contents in bPVP and sPVP were on par with those of collagen. The elastin contents were somewhat lower/higher in the acid hydrolysis than those of alkaline treatment. The elastin contents in bPcm and SIS however were significantly lower than those in bPVP and sPVP with either the acid hydrolysis or alkaline treatment (*p* < 0.05). The ratio of elastin to collagen is 1.18 or 1.46 in bPVP and 1.06 or 1.25 in sPVP due to the different methods. The ratio of elastin to collagen is 0.02 or 0.09 in bPcm and 0.17 or 0.11 in SIS (Table 2).

**TABLE 2:** Dry weights of elastin and collagen in the tissues.

Unit: μg/mg	Elastin-CI	Elastin-IS	Collagen	E-CI/Col	E-IS/Col
bPVP	252.1 ± 30.1	361.4 ± 38.6	216.7 ± 27.9	1.18 ± 0.24	1.46 ± 0.51
sPVP	247.8 ± 27.3	325.7 ± 35.9	237.3 ± 29.1	1.06 ± 0.21	1.25 ± 0.39
bPcdm	9.3 ± 1.8^*^	54.8 ± 9.2^*^	410.3 ± 59.8^*^	0.02 ± 0.01^*^	0.09 ± 0.08^*^
SIS	61.2 ± 9.5^#^	19.5 ± 4.3^#^	373.5 ± 43.6^#^	0.17 ± 0.04^#^	0.11 ± 0.05^#^

*: *p<*0.05 in comparison with the elastin in bPVP.

#: *p<*0.05 in comparison with the elastin in sPVP.

bPVP, bovine pulmonary visceral pleura; sPVP, swine pulmonary visceral pleura; bPcdm, bovine pericardium. SIS, swine small intestine submucosa; elastin-CI, converted insoluble to soluble elastin to measure; elastin-IS, insoluble elastin was measured; E-CI/Col, ratio of elastin-CI to collagen; E-IS/Col, ratio of elastin-IS to collagen.

### Immunofluorescence Microscopy

**FIGURE 2:**
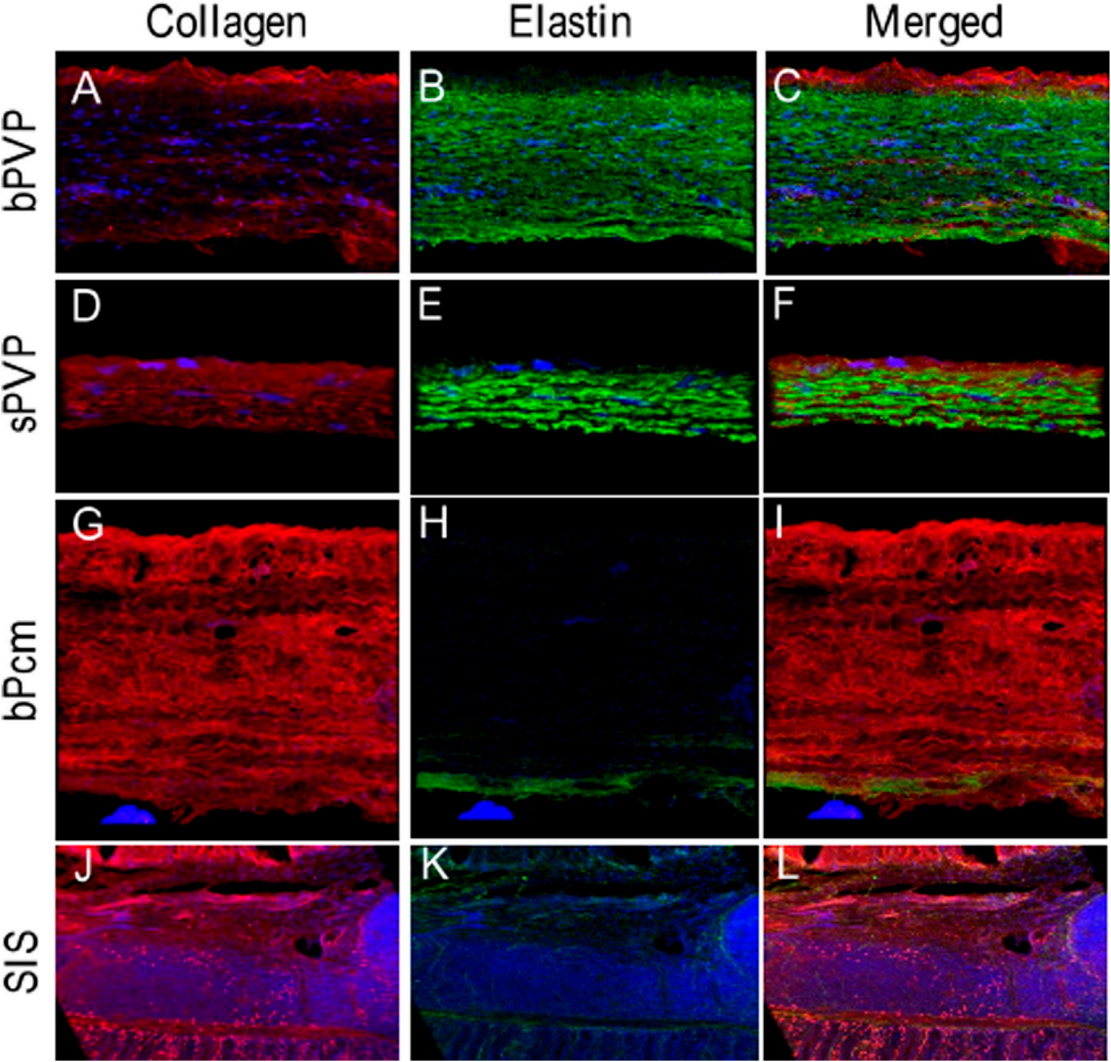
Collagen and elastin fibers in bPVP, sPVP, bPcm, and SIS. **(A–C)** Collagen, elastin, and merged collagen and elastin in bPVP, respectively. **(D–F)** Collagen, elastin, and merged collagen and elastin in sPVP, respectively. **(G–I)** Collagen, elastin, and merged collagen and elastin in bPcm, respectively. **(J–L)** Collagen, elastin, and merged collagen and elastin in SIS. Red: anti-collagen; green: anti-elastin; blue: nuclei. Objective: ×60.

Collagen and elastin in the various tissues were visualized under immunofluorescence microscopy. The collagen in bPVP and sPVP was denser on the mesothelial basal lamina and lesser in the middle and parenchyma sides (Figures 2A,D). The elastin was dense throughout the thickness of both bPVP and sPVP as robustly visualized (Figures 2B,E). The fluorescence images of collagen and elastin in bPVP and sPVP were merged and are shown in Figures 2C,F. The collagen in bPcm was evenly distributed throughout the thickness of the section, and the collagen fibers were easily observed (Figure 2G). The elastin fibers in bPcm were sparsely found in the middle and a little robust in the posterior (Figure 2H). The collagen was unevenly distributed in the SIS, that is, it was denser in the adjacent regions toward mucosa and muscularis externa than that in the middle region (Figure 1J). The elastin fibers were sparsely observed in SIS (Figure 2K). The fluorescence images of collagen and elastin in bPcm and SIS were merged and are represented in Figures 1I,L.

### Uniaxial Mechanical Property

**FIGURE 3:**
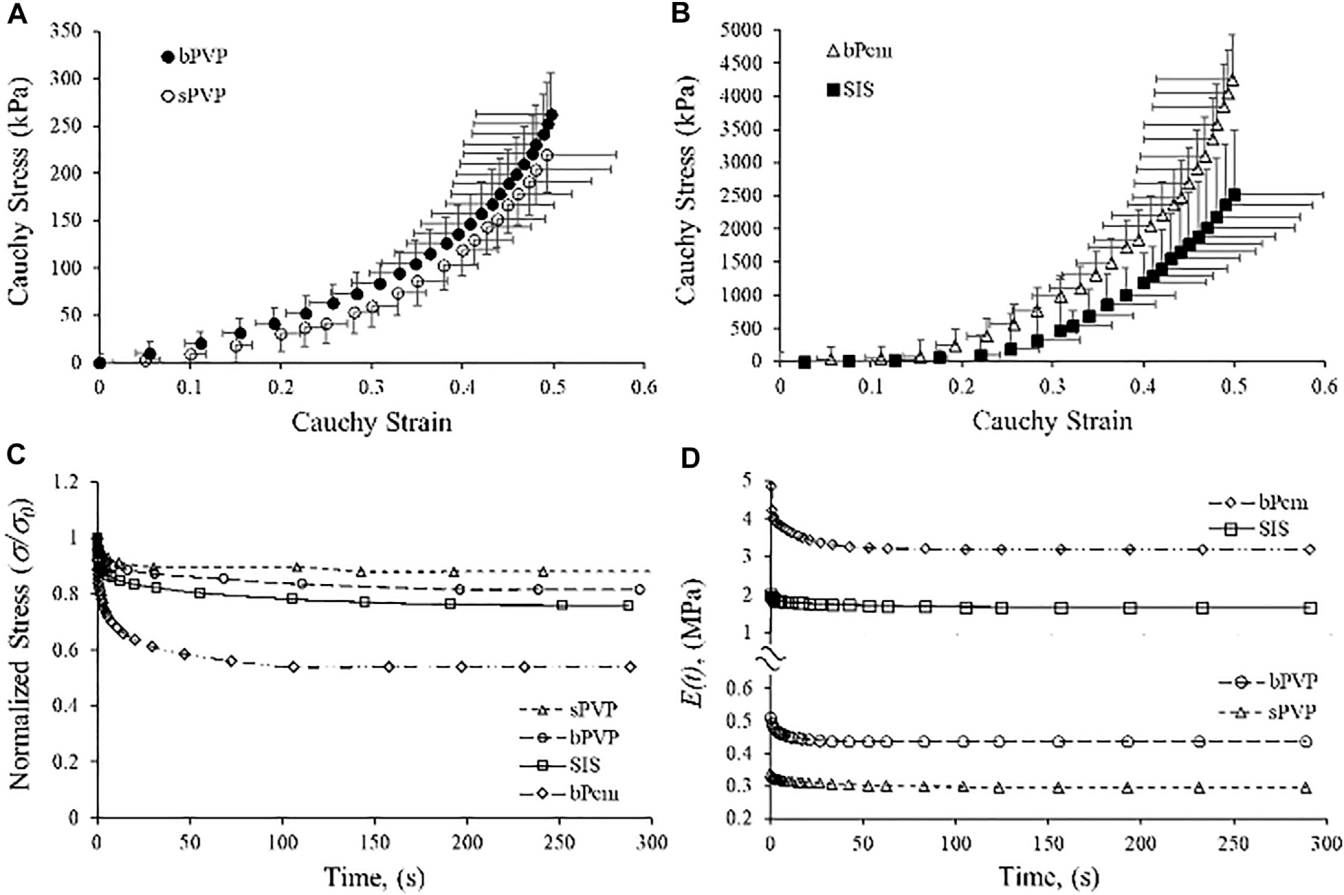
Relationship of Cauchy stress-to-strain and stress relaxation of bPVP, sPVP, bPcm, and porcine SIS. **(A)** The relations of bPVP and sPVP. **(B)** The relations of bPcm and SIS. **(C)** Typical curves for stress relaxation of sPVP, bPVP, SIS, and bPcm. **(D)** Time-dependent modulus *E*_*relax*_(*t*) for sPVP, bPV, SIS, and bPcm.

The Cauchy stress–stretch relations and stress relaxation of the PVP, SIS, and bPcm are represented in Figure 3. The stress–strain relation of the sPVP was close to the bPVP where the slope increased from 0 to 0.5 when stretched (Figure 3A). The stress of the bPVP at a strain of 0.49 was 262.8 ± 48.3 kPa, which was not significantly larger than 219.7 ± 38.9 kPa of the sPVP (*p >* 0.05). The stress–strain relations of the bPcm and SIS are represented in Figure 3B. The stress–strain relation of the bPcm was larger than the SIS in the range of strain 0–0.5 (*p <* 0.05, two-way ANOVA). The stress of the bPcm at strain 0.49 was 4,262.8 ± 657.3 kPa, which was significantly larger than the 2,516.3 ± 976.1 kPa of the SIS (*p <* 0.05). The stress of the SIS at strain 0.49 was approximately 10-fold of the stresses of the bPVP and sPVP.

The typical curves for stress relaxation are represented in Figure 3C, which differed for sPVP, bPVP, SIS, and bPcm. The time-independent elastic modulus (*E*_*0*_), the modulus and relaxation–time pairs for a fast-response element (*E*_*1*_ and *t*_*1*_), and the modulus and relaxation–time pairs for a slow-response element (*E*_*2*_ and *t*_*2*_) are represented in Table 2. The goodness-of-fit parameter for the fitted curves (*R* ^*2*^) indicates a satisfactory representation of the data (Table 3). The viscoelastic parameters of sPVP and bPVP are significantly different from bPcm and SIS (*p* < 0.05). The *E*_*relax*_(*t*) for sPVP, bPVP, SIS, and bPcm is represented in Figure 3D, which shows that *E*_*relax*_(*t*) for bPcm and SIS is one order magnitude higher than that for sPVP and bPVP.

**TABLE 3:** Viscoelastic parameters of the tissues’ best fit with the Maxwell–Wiechert model.

	*E*_*0*_ (MPa)	*E*_*1*_ (MPa)	*E*_*2*_ (MPa)	*τ*_*1*_ (s)	*τ*_*2*_ (s)	*R* ^*2*^
sPVP	0.31 ± 0.11^*#^	0.02 ± 0.01^*#^	0.03 ± 0.01^*#^	2.57 ± 1.65^*#^	48.3 ± 18.5	0.99 ± 0.01
bPVP	0.44 ± 0.16^*#^	0.04 ± 0.02^*#^	0.04 ± 0.02^#^	1.73 ± 1.15^#^	47.3 ± 12.6	0.96 ± 0.04
SIS	1.7 ± 0.8	0.18 ± 0.15^#^	0.18 ± 0.17^#^	1.57 ± 0.15	46.3 ± 10.4	0.99 ± 0.01
bPcm	3.2 ± 1.8	0.72 ± 0.43	0.74 ± 0.46	0.67 ± 0.21	47.7 ± 11.2	0.99 ± 0.01

* *p* < 0.05 in comparison with SIS; #, *p* < 0.05 in comparison with bPcm.

*E*_*0*_: the time-independent elastic modulus. *E*_*1*_ and *τ*_*1*_: the modulus and relaxation–time pairs for a fast-response element.

*E*_*2*_ and *τ*_*2*_: the modulus and relaxation–time pairs for a slow-response element. *R* ^*2*^: the goodness-of-fit parameter for the fitted curves.

### *In Vitro* Cytotoxicity

**FIGURE 4:**
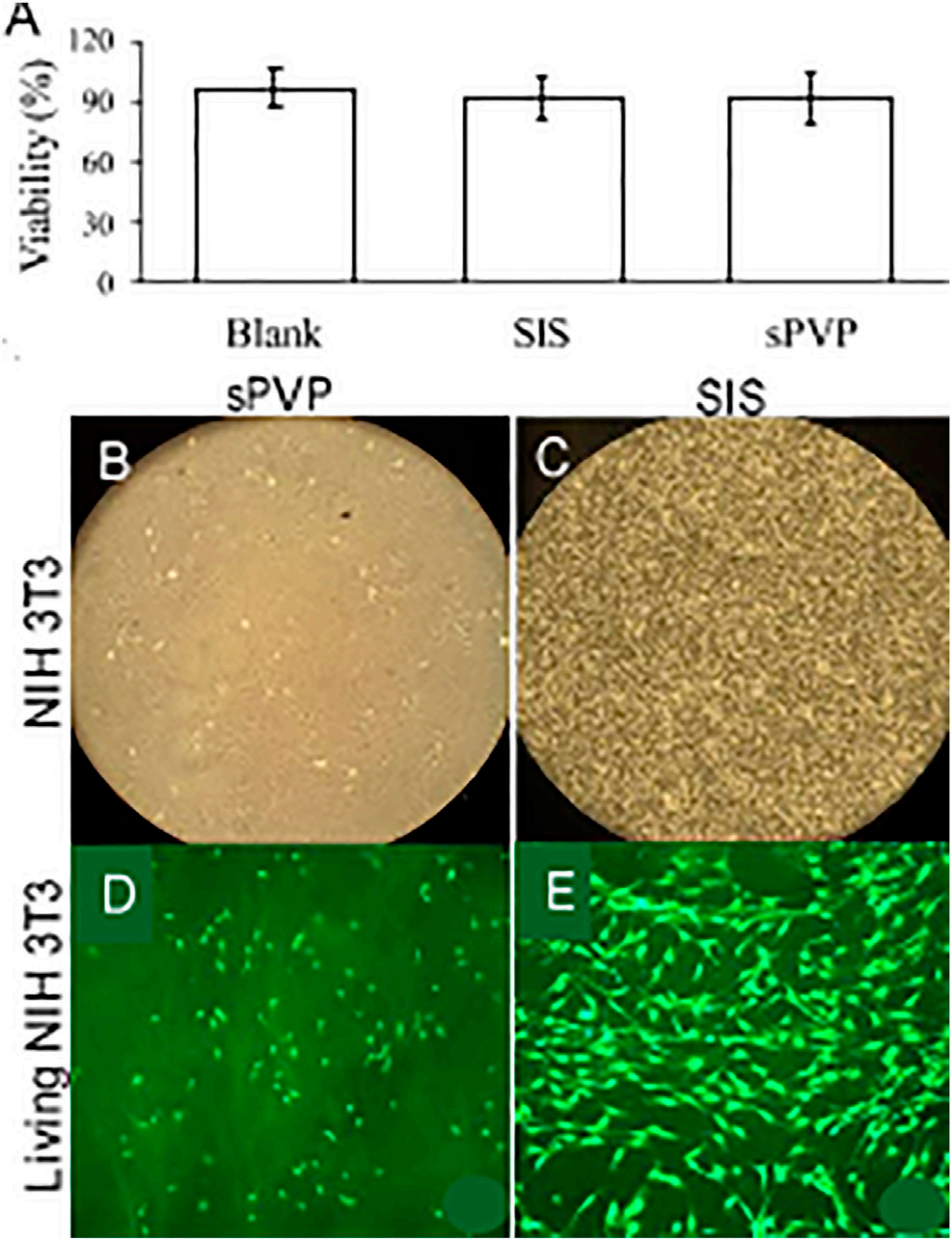
*In vitro* cytotoxicity of sPVP in comparison with SIS. **(A)** Cellular viability (%). **(B–C)** NIH 3T3 cells on sPVP and SIS, respectively. Objective: ×4. **(D–E)** Calcein marked (green) NIH 3T3 cells on the PVP and SIS, respectively. Objective: ×60.

Cell viability (%) is represented in Figure 4A. Fibronectin-coated wells served as blanks of *in vitro* cytotoxicity. Cell viability on SIS and sPVP slightly decreased in the 5-day experimental period in comparison with control (*p* > 0.05), that is, a negligible cytotoxicity in SIS and sPVP. Figures 4B,C show global views of the cells on the SIS and the PVP for 5 days, respectively. The living cells (green, calcein AM labeled) on the SIS and PVP are shown in Figures 4D,E, respectively.

### *In Vivo* Biocompatibility

**FIGURE 5:**
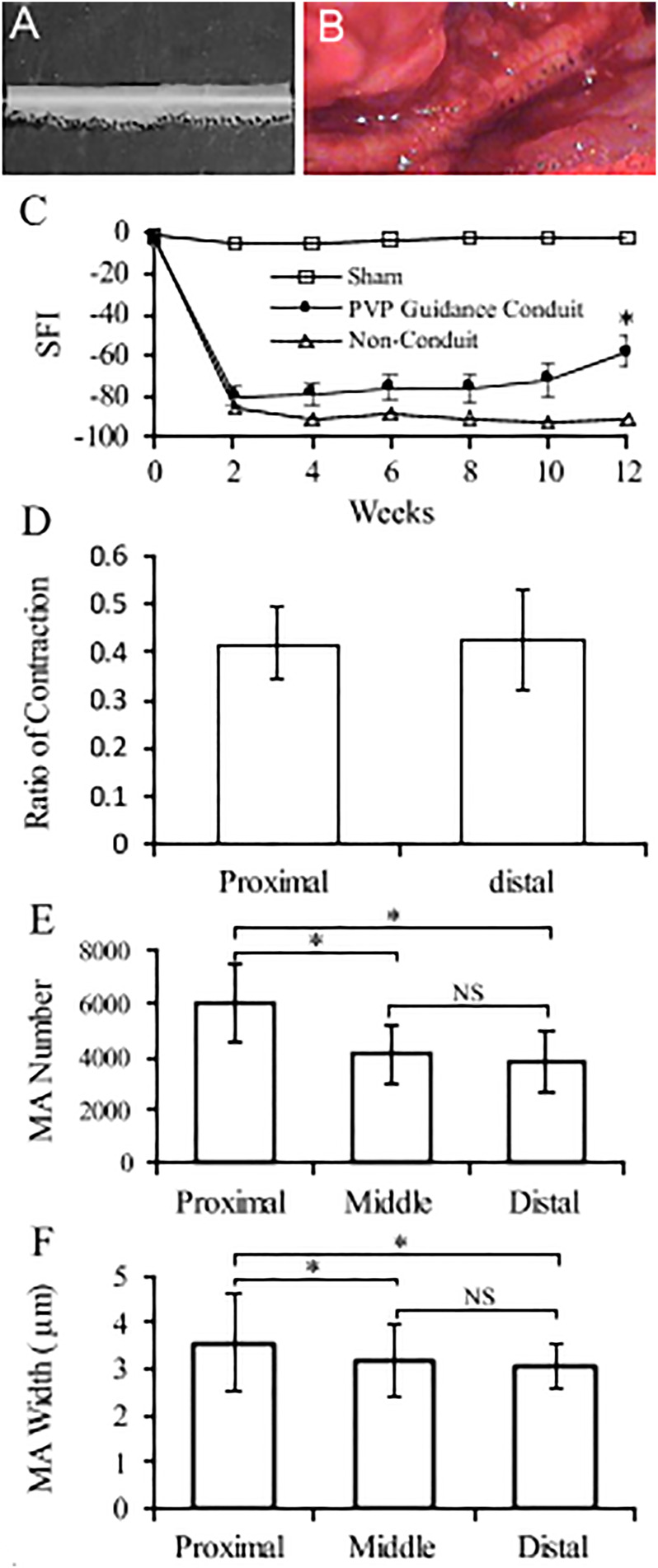
Nerve conduit guidance and parameters of nerve regeneration in sciatic model of rats. **(A)** An example of conduit guidance before implantation. **(B)** An example of conduit guidance at postop 12 weeks. **(C)** Biweekly changes of SFI in rodent sciatic nerve. Sham: non-injury to the sciatic nerve during the sciatic nerve exposure and skin closure. PVP guidance conduit: The sPVP nerve guidance conduit implanted when the sciatic nerve excised. Non-conduit: no guidance conduit implanted when the sciatic nerve was excised. **(D)** Ratios of the ipsilateral tetanic contractions of sPVP nerve guidance conduit to contralateral tetanic contractions of the gastrocnemius muscle. Proximal and distal: a bipolar electrode was placed at the proximal or distal end of the sPVP nerve guidance conduit, respectively. **(E)** The number of myelinated axons in the proximal, middle, and distal regions of the sPVP nerve guidance conduit, respectively. **(F)** The average width of myelinated axons in the proximal, middle, and distal regions of the sPVP nerve guidance conduit, respectively. SFI: sciatic function index. MA: myelinated axons. *: *p* < 0.05. NS: *p* > 0.05.

**FIGURE 6:**
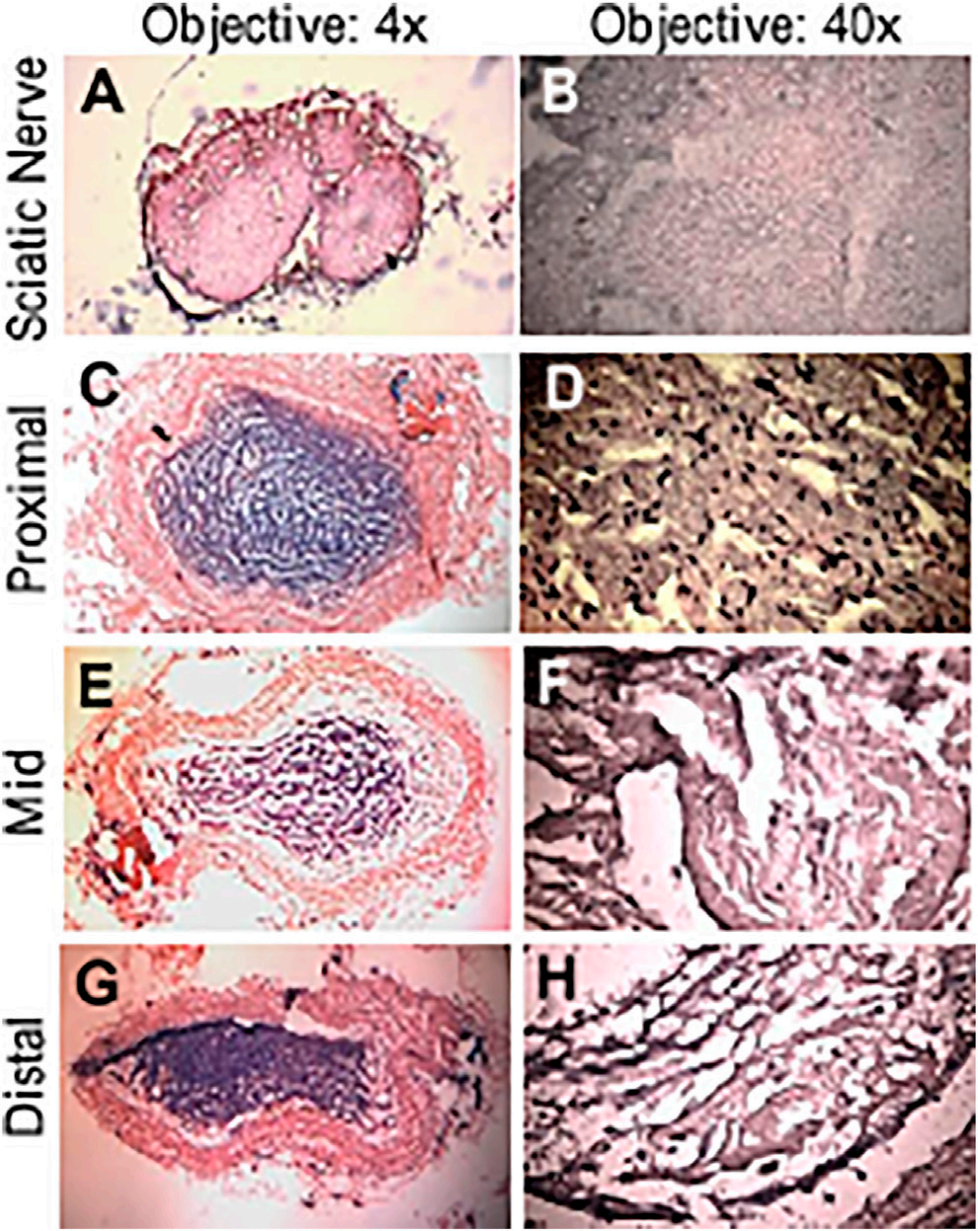
HE of the sPVP nerve guidance conduit in sciatic model of rats. **(A)** Sciatic nerve fibers at low magnification (objective: ×4). **(C, E, and G)** The proximal, middle, and distal regions of the sPVP nerve guidance conduit at low magnification (objective: ×4), respectively. **(B)** Sciatic nerve fibers at high magnification (objective: ×40). **(D, F, and H)** The proximal, middle, and distal regions of the sPVP nerve guidance conduit at high magnification (objective: ×40), respectively.

**FIGURE 7:**
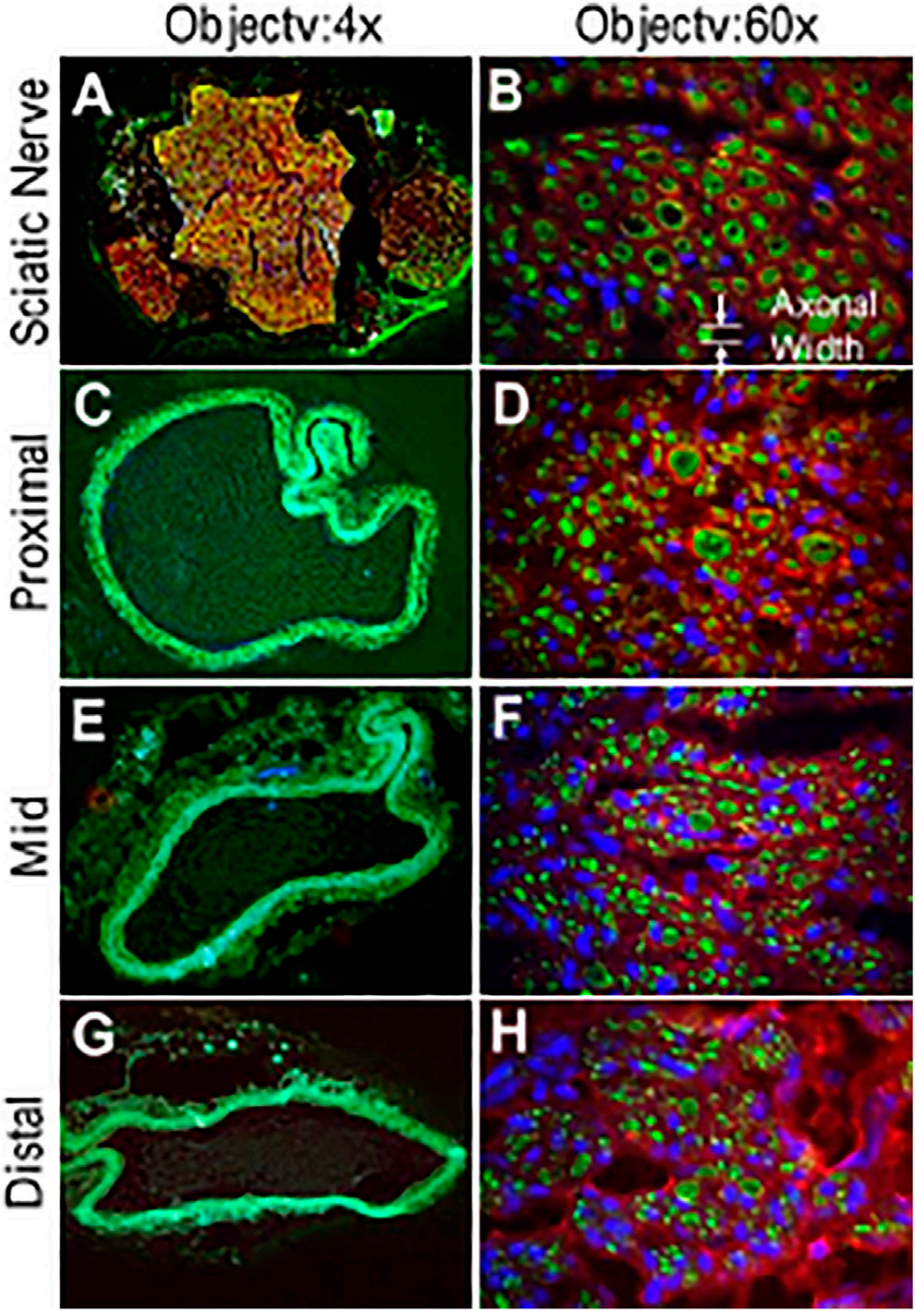
Immunofluorescence images of the sPVP nerve guidance conduit in sciatic model of rats. **(A)** Sciatic nerve fibers at low magnification (objective: ×4). **(C, E, and G)** The proximal, middle, and distal regions of the sPVP nerve guidance conduit at low magnification (objective: ×4), respectively. (**B)** Sciatic nerve fibers at high magnification (objective: ×60). **(D, F, and H)** The proximal, middle, and distal regions of the sPVP nerve guidance conduit at high magnification (objective: ×60), respectively. Red: anti-MBP (myelin basic proteins); green: anti-NFLP (neurofilament light polypeptide); blue: nuclei of cells. Objective: ×60. The white lines and arrows indicate the measurement of axonal width.

An example of conduit guidance before implantation and postop 12 weeks is shown in Figures 5A,B. The SFI slightly varied between −2 and −7 through postoperative 12 weeks (Figure 5C), when the sciatic nerve was not injured but only exposed (sham), and then the skin was closed (Wang et al., 2015b; Ma et al., 2014). The SFI decreased from −2.9 ± 4.1 for the non-injury of the sciatic nerve to −80.1 ± 5.1 for the transection of the sciatic nerve. The SFI after implanted PVP guidance conduit was slightly recovered to −58.9 ± 7.6 at postoperative 12 weeks (*p* < 0.05, Figure 5C). The recovery in SFI indicates that the sciatic nerve function was improved by the PVP guidance conduits. In contrast, the SFI slightly varied between −85 and −95 through postoperative 12 weeks (Figure 5C), when the sciatic nerve (∼10 mm) was excised and no guidance conduit was implanted for the connection of two stumps (Ma et al., 2014; Wang et al., 2015b). The tetanic contraction of the gastrocnemius muscle (contractility) was represented as the ratio of the force in the ipsilateral sPVP nerve guidance conduit to the contralateral non-traumatic sciatic nerve (Figure 5D). There was no difference of the ratio for stimulation at proximal or distal ends of the sPVP nerve guidance conduit (*p* > 0.05). The ratio of wet gastrocnemius muscle mass is 0.50 ± 0.035. Based on immunofluorescence microscopy, the axonal numbers and width of myelinated axons (MA) were analyzed and are represented in Figures 5E,F. Histological analysis showed that the sPVP nerve guidance conduit was filled with regenerative tissues (Figures 6C–H), and Figures 6A,B show sciatic nerve fibers. The positive fluorescence signals of neurofilament light polypeptide and myelin basic proteins in the sPVP nerve guidance conduit confirm that the regeneration of axons bridges the proximal and distal stumps of the sciatic nerve (Figures 7C–H), and Figures 7A,B show sciatic nerve fibers.

**FIGURE 8:**
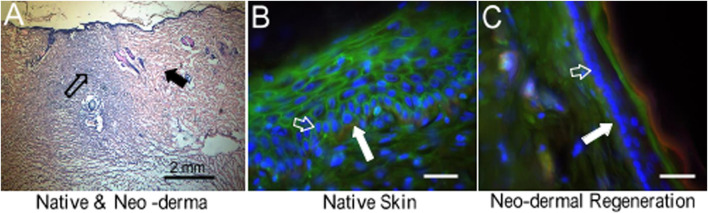
HE staining and fluorescence microscopic images to represent skin regeneration after the wound created in rats. **(A)** HE stain of neo-dermal regeneration (black hollow arrow) and native skin (black solid arrow). Objective: ×4. **(B) N**ative skin. **(C)** Neo-dermal regeneration filled in the wound. Objective: ×40. Red: green: sialic acid. Blue: nuclei. Collagen type IV. Bar: 50 μm. White solid arrows indicate collagen type IV in the basal lamina. White hollow arrows indicate keratinocytes which were proliferating during skin regeneration.

In a skin substitute with sPVP, no infection or complications were observed in the rats postoperation. The neo-dermal regeneration under the sPVP had filled the wound at 4 weeks postoperative (Figure 8A). The immunofluorescence microscopy represented keratinocyte lining on the basal lamina of native skin (Figure 8B). Neo-keratinocyte lining on the basal lamina (collagen IV, red) was observed in the regenerative skin (Figure 8C), which implicates the epidermal layer was formed in the regenerative skin (Figure 8C). Based on immunofluorescence microscopy, the numbers of keratinocyte in unit length (mm) were 92 ± 31/mm for native skin and 106 ± 37/mm for neo-derma (*p* > 0.1).

## Discussion

We found that the bPVP and sPVP are composed of abundant collagen and elastin. Specifically, the elastin contents are approximately equal to the collagen in the bPVP and sPVP. The thick and dense fibers of elastin were robustly observed in immunofluorescence microscopy. Therefore, the bPVP and sPVP can be defined as an elastin- and collagen-based ECM in comparison with primarily collagen-based ECM biomaterials such as swine SIS and bovine pericardium. The biomechanical analyses of stress–strain and stress relaxation confirm that the compliance of bPVP and sPVP is greater than bPcm and SIS. Furthermore, we found low cytotoxicity of PVP in the *in vitro* study and excellent biocompatibility in the *in vivo* implantation of the PVP as nerve guidance conduit and skin substitute.

### Strengths of Elastin–Collagen ECM

Collagen and elastin are known as the critical ECM and regulate cellular proliferation and functionalization. Therefore, ECM is a desirable biomaterial in the reconstruction of organs or tissues, such as bPcm and SIS, which are known as collagen-based ECM and diversely utilized in the clinic. In the present study, we verified that the contents of elastin and collagen are approximately equivalent in the PVP. Therefore, we introduce PVP as an elastin- and collagen-based ECM, and expect that both collagen and elastin can enhance cellular proliferation and functionalization in organ or tissue reconstruction. It is well accepted that a biological material must be strong, flexible, and durable. Collagen-based ECM has manifested its excellent characteristics. Collagen may be further cross-linked to resist MMPs’ degradation. Elastin fibers in the PVP further enhance the flexibility and durability of collagen-inherent characteristics. It is known that elastin fiber is extensively cross-linked in ECM, which renders elastin for the protein’s insolubility and contributes to its longevity (Mecham, 2018). Shapiro et al. estimated the life span of elastin using aspartic acid racemization at 14°C that turnover to be ∼80 years in humans (Shapiro et al., 1991). Studies using sensitive immunological techniques to measure elastin peptides in the blood or desmosine cross-links excreted in the urine suggest that <1% of the total body elastin pool turns over in a year (Starcher, 1986; Mecham, 2018). Another factor contributing to the longevity of mature elastin is its relative resistance to proteolysis. Since there are few lysine or arginine residues in the fully cross-linked protein, and few amino acids with large aromatic side chains, elastin is not degraded by trypsin- or chymotrypsin-like proteases. In the present study, the immunofluorescence microscopy reveals that the thick fibers of elastin densely distribute through the PVP (Figures 2B,E). The thick fibers of elastin may retain the elastic property of the PVPs for long term.

### Mechanical Mismatch

Mechanical property is a critical feature of biomaterials. Mechanical and structural mismatches between the biomaterial and native tissue may alter the local mechanical environment and contribute to abnormal growth and remodeling (Zilla et al., 2007; Shi and Tarbell, 2011). It is known that mechanical mismatch is a cause of poor performance of a vascular graft (Zilla et al., 2007; Sánchez et al., 2018). The compliance mismatch of the graft with an adjacent blood vessel can result in altered blood flow, platelet activation, and thrombus formation (Malek et al., 1999; Zilla et al., 2007). The reductions of intramural stress may mitigate propensity for mineralization such as in bioprosthetic heart valves (Munnelly et al., 2012). Therefore, the similarity of mechanical and structural properties of biomaterials and host is critical for successful tissue reconstruction (Zilla et al., 2007).

In this study, we verified that the incremental moduli in stress–strain curves and relaxation moduli in the Maxwell–Wiechert model of sPVP and bPVP were approximately one-tenth of bPcm and SIS. The prostheses made of sPVP and bPVP may mitigate some of the complications due to mechanical mismatch with biological tissues. The mechanical properties of fibrous tissues stem from their constituents, that is, collagen and elastin fibers (Lokshin and Lanir, 2009; Wang et al., 2015a). A proper content of elastin and collagen is necessary to render ideal mechanical properties for a biomaterial. Previous studies indicated that the PVP consists of more elastin (∼25% dry weight) and maintains one-fifth as much collagen as the parenchyma, which serves the role of PVP mechanics (Oldmixon and Hoppin, 1984; Starcher, 1986; Fraser et al., 2005). The PVP has been viewed as a complex structure that adds to tissue disparity (Michailova, 1997). The immunofluorescence microscopy reveals the microstructure of PVP tissues, which is consistent with the macroscopic mechanical responses, where the stress–strain curve of the PVP becomes non-linear at a larger stretch ratio with higher stress level than at the lower loads as shown in Figure 3A. Specifically, the PVP microstructure and mechanical properties indicate that it is a highly compliant material. Hence, the PVP may be different for bioprosthetic applications, where the mechanical compliance needs to be adopted.

### *In Vivo* Biocompatibility

There is no doubt that collagen and elastin have excellent biocompatibility because collagen and elastin are the major constituents of ECM and provide the mechanical environment of cell proliferation and biological functions. Therefore, it is not surprising that the cytotoxicity of the PVP is as low as that of SIS (Figure 4). It is well known that the *in vivo* experiment is the proper test for biocompatibility of biological prosthesis. In fact, the *in vivo* experiment is also an appreciated test for mechanical coupling of biomaterials to native tissues. In the present study, we designed *in vivo* nerve conduit guidance and skin substitute for the evaluation of the biocompatibility and mechanical coupling of the PVP. Although the autologous nerve graft remains the gold standard treatment for repair of peripheral nerve defects (Schmidt and Leach, 2003; Daly et al., 2012), there is a limitation of tissue harvest numbers and dimensions where extensive reconstruction is required (Schmidt and Leach, 2003). Nerve guidance conduit is used as an alternative autologous nerve graft for peripheral nerve repair. Although biological and synthetic materials are used for nerve guidance conduit, axonal regenerative capacity in existing nerve guidance conduit is often incomplete and functional recovery is limited. Novel biomaterials for nerve guidance conduit are needed to improve the functional and structural outcomes in nerve repair. The PVP mechanical compliance allows the PVP nerve guidance conduit to assimilate into adjacent tissues. Furthermore, both collagen and elastin are ameliorative to support nervous regeneration (Waitayawinyu et al., 2007; Whitlock et al., 2009; Hsueh et al., 2014). In the rodent model, we demonstrate that the PVP nerve guidance conduit is suitable for peripheral nerve repair/reconstruction.

The skin covers the body to provide protection and receive sensory stimuli from the external environment. Minor wounds can heal well, but severe wounds need rapid repair to prevent scar invasion and water loss. Although skin transplantation is the “gold standard” for repair/reconstruction, artificial skin is frequently used to repair wounds when an autologous donor is not available. Typical artificial skin is composed of collagen to promote regeneration of skin and accommodate epidermal cell migration and proliferation for re-epidermalization on the wound. Considering skin is an elastin-abundant organ, the abundant elastin in the PVP skin substitute can minimize the mechanical mismatch between the substitute and native skin. The abundant elastin can suppress the overgrowth of fibroblast during neo-dermal regeneration to mitigate scar formation because it has been demonstrated that scarring could be mitigated by controlling fibroblast plasticity (Plikus et al., 2015; Chan and Longaker, 2017). We demonstrated in rodent models that the PVP skin substitute promoted wound healing, and scars did not form during the period of the study.

### Study Limitation

We employed acid hydrolysis plus TPPS dye and alkaline treatment for the analyses of elastin contents in various tissues. The variations of elastin contents in the same tissue require discussion. In acid hydrolysis, the thick fibers of insoluble elastin may diminish the efficacy to convert insoluble elastin to soluble elastin. Therefore, the elastin contents may be underestimated in the PVP since the insoluble elastin may not be completely converted to soluble elastin. In alkaline treatment, the microfibrils of insoluble elastin may be rinsed out from loose ECM such as the SIS, which may result in underestimation of insoluble elastin in the SIS. The mechanical property of the PVP in comparison with the bPcm and SIS may provide additional parameters to verify an abundant elastin in the PVP.

## Conclusion

The abundant collagen and elastin contents in sPVP and bPVP confer the merits of an elastin- and collagen-based ECM, including the mechanical compliance, cytotoxicity, biocompatibility, and regulation of cellular proliferation. I*n vitro* studies indicate that the PVP has minimal cytotoxicity. *In vivo* studies indicate excellent PVP biocompatibility and minimal inflammation. Hence, the PVP is a suitable biological material for the repair/reconstruction of soft tissues due to its abundant collagen and elastin contents, structural characteristics, and mechanical compliance.

## Data Availability

The original contributions presented in the study are included in the article/supplementary material; further inquiries can be directed to the corresponding author.
